# Exploring memory synchronization and performance considerations for FPGA platform using the high-abstracted OpenCL framework: Benchmarks development and analysis

**DOI:** 10.1371/journal.pone.0301720

**Published:** 2024-05-13

**Authors:** Abedalmuhdi Almomany, Amin Jarrah, Muhammed Sutcu

**Affiliations:** 1 Department of Electrical & Computer Engineering, Gulf University for Science & Technology, Kuwait, Kuwait; 2 Department of Computer Engineering, Hijjawi Faculty for Engineering Technology, Yarmouk University, Irbid, Jordan; 3 Department of Engineering Management, Gulf University for Science & Technology, Kuwait, Kuwait; Lanzhou University of Technology, CHINA

## Abstract

A key benefit of the Open Computing Language (OpenCL) software framework is its capability to operate across diverse architectures. Field programmable gate arrays (FPGAs) are a high-speed computing architecture used for computation acceleration. This study investigates the impact of memory access time on overall performance in general FPGA computing environments through the creation of eight benchmarks within the OpenCL framework. The developed benchmarks capture a range of memory access behaviors, and they play a crucial role in assessing the performance of spinning and sleeping on FPGA-based architectures. The results obtained guide the formulation of new implementations and contribute to defining an abstraction of FPGAs. This abstraction is then utilized to create tailored implementations of primitives that are well-suited for this platform. While other research endeavors concentrate on creating benchmarks with the Compute Unified Device Architecture (CUDA) to scrutinize the memory systems across diverse GPU architectures and propose recommendations for future generations of GPU computation platforms, this study delves into the memory system analysis for the broader FPGA computing platform. It achieves this by employing the highly abstracted OpenCL framework, exploring various data workload characteristics, and experimentally delineating the appropriate implementation of primitives that can seamlessly integrate into a design tailored for the FPGA computing platform. Additionally, the results underscore the efficacy of employing a task-parallel model to mitigate the need for high-cost synchronization mechanisms in designs constructed on general FPGA computing platforms.

## 1. Introduction

**Fig 1:**
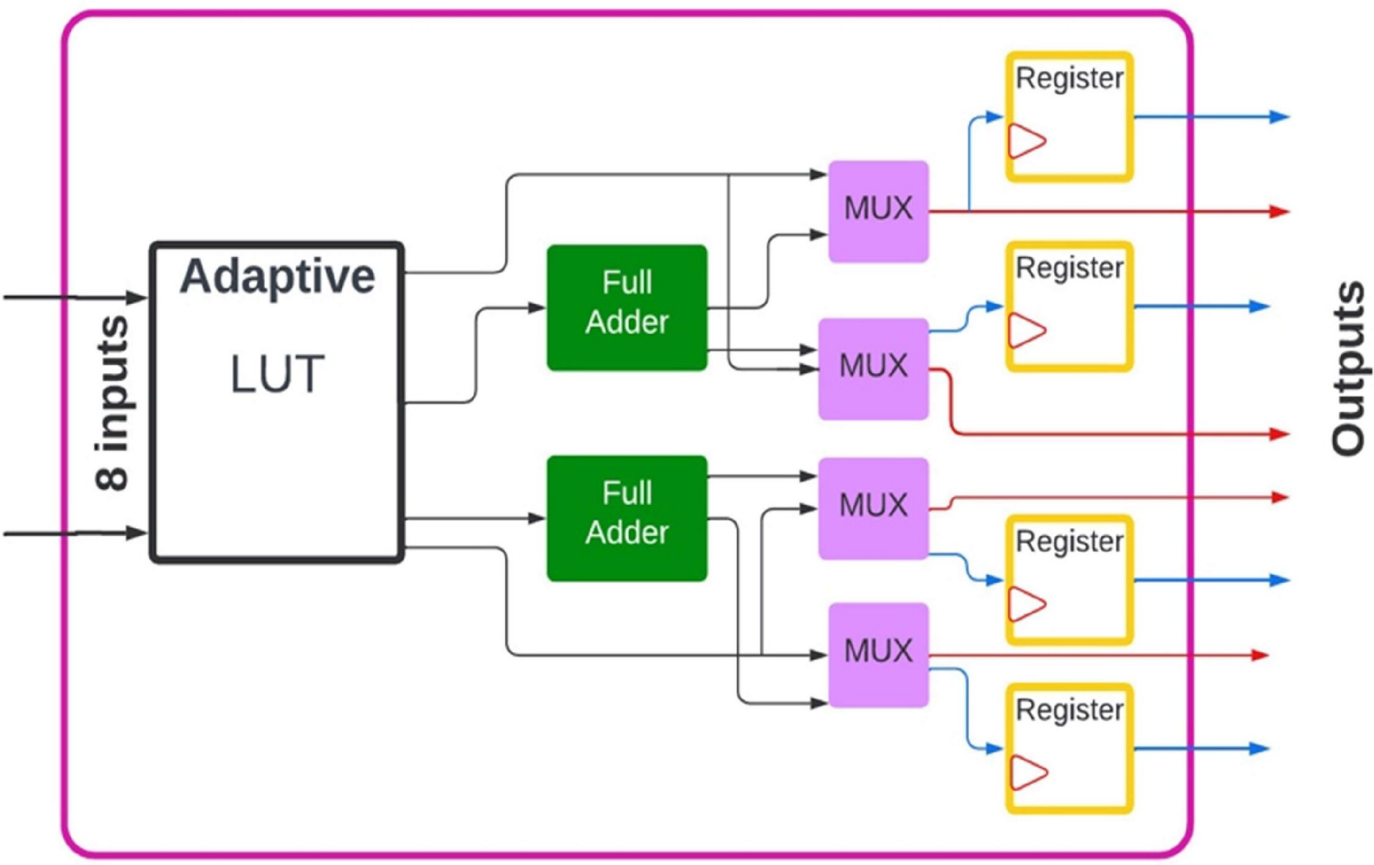
Adaptive logic module (ALM) block diagram.

A field programmable gate array (FPGA) is an integrated circuit that programmers can configure many times to achieve their goals [1, 2]. FPGAs include many low-level operations, such as shifts and additions. Usually, the Intel FPGA incorporates several resources, such as RAM blocks and DSPs, to perform various complex arithmetic functions and look-up tables (LUTs) [2]. LUTs are also used to implement several functions (Fig 1). However, multiple LUTs can be combined to implement more complex functions. The DE5 (Stratix V) FPGA device is used in the present study. The adaptive logic module (ALM) resource allows a wide range of functions to be implemented efficiently. Each ALM contains several function units. The block diagram for the ALM is shown in Fig 1 [3]. FPGA as a reconfigurable architecture provides better reconfiguration in which bit-level configuration is performed [4–7].

The flexible parallel hardware architecture is guaranteed by FPGA technology. It includes many logic components, such as adders, multipliers, and comparators. It also includes a lot of DSPs (Digital Signal Processors), LUTs (Look Up Tables), clocks, configurable I/O, memories, and wired connections between these components. Because these components operate concurrently, allowing for a large amount of computation to be done independently at once, we can achieve a high level of parallelization with this FPGA implementation [8–10]. Many semiconductor companies, including Xilinx, Altera, Actel, Lattice, Quick Logic, and Atmel, produced and improved FPGA.

Three types of FPGA-based spatially reconfigurable computing environments are now commercially available. They include commodity FPGA-based accelerator cards, stand-alone SOPC environments, and cloud-based spatially reconfigurable platforms. Commodity FPGA-based accelerator cards are the most common commercially available spatially reconfigurable computing environment and were chosen as the computing environment for this research. These cards are designed to be incorporated within a standard CPU-based computing system as an add-on low-profile PCIe-based daughter card. They incorporate one or more high-end FPGAs and significant amounts of multi-banked DDR SDRAM physical memory (8 GB to 32 GB), which are local to the card. The cards often also contain high speed network ports and flash memory that can be used to load default configurations within the FPGAs. Stand-alone SOPC configurations are also quite prevalent at the time of this writing. SOPC configurations also include high-end FPGAs that often contain built-in embedded CPU processing cores. SOPC configurations also contain varying amounts of DDR SDRAM physical memory and a host of I/O interfaces. SOPC configurations differ primarily from accelerator cards in that they are not designed to augment an existing CPU system [11].

FPGA platforms are now becoming available on the cloud [12]. FPGA-based resources are accessible using the OpenStack virtual machine environment, which provides tools for cloud resource management [13, 14]. In a related study, a framework that integrated Xilinx FPGAs into the cloud based on OpenStack showed great efficiency and scalability upon hosting multiple processes and VMs [15]. FPGAs are also accessible as an F1 compute instance on the Amazon Elastic Cloud, where each instance contains up to eight FPGAs [16]. These instances could be used effectively to create a customized user’s design in a wide range of commercial and scientific applications.

Commodity FPGA-based technology has several issues, though, which must be carefully considered. One important issue is that while it is possible to create specialized functional units and data paths that closely mirror the structure of the application, the FPGA resources that are required are usually only a fraction of those required to implement the application in its most optimized form. Thus, the intelligent time sharing of these resources is mandatory and is the system-wide focus of what is a very complex optimization problem. The time it takes to configure an FPGA is large compared to the time taken to perform a base operation. The reconfiguration time for large FPGAs can be in the order of seconds, whereas the internal clock speed can be greater than 300 MHz. This means that internal FPGA resource trade-offs may have to be made that will decrease the utilization and increase time sharing to reduce the number of FPGA reconfigurations required. Another possibility is to utilize partial reconfigurability, which is supported by most modern FPGAs. Partially reconfigurable devices allow for the logic functionality of a subsection of its programmable resources to be reconfigured without interrupting the operation of the other portion of the reconfigurable logic. Unfortunately, this feature is often poorly utilized. Another major issue is the time it takes to synthesize a design. The fine-grain complexity of FPGAs can result in extremely long design compilation times, which can take hours or days to complete. This problem is most apparent when the FPGA-based resources needed by the application get close to the actual resources that are present on the system. It becomes imperative in such cases that the high-level design environment allow for the functionality of the design to be verified quickly before it goes through this lengthy process. Fortunately, high-level synthesis environments, such as the OpenCL support an emulator mode where emulation can be performed on the CPU. Still, this constraint precludes the use of just-in-time compilation techniques that are possible in GPU and some CPU applications. This means all modules that are to be executed on the FPGA must therefore be progenerated in an offline manner [11].

To implement the proposed design on a general FPGA computation platform, one can employ languages such as Verilog, VHDL, or other supportive languages like System-C. However, opting for VHDL or Verilog for the suggested implementation entails writing numerous lines of code, even for relatively simple tasks. In contrast, accomplishing a similar task using OpenCL requires only a few lines of code. Programmers who target Hardware Description Languages (HDLs) must possess substantial experience with the underlying hardware, whereas OpenCL abstracts away these hardware details.

OpenCL is an open computing language used to harness the benefits of multi-processing elements. The wide variety of platforms that can be used for OpenCL makes it an attractive choice for heterogeneous systems in which several computations can be distributed among different computation architecture elements [10]. The OpenCL code written to run on the FPGA is implemented as a kernel, and the kernel code is compiled using the Intel offline compiler (IOC). The kernel could be executed with one or multiple items (threads) [11]; the choice depends on the code characteristics, and the goal is to achieve the highest degree of parallelism. The OpenCL standard naturally enables the ability to specify parallel algorithms to be implemented on FPGAs, at a far higher degree of abstraction than hardware description languages (HDLs) like VHDL or Verilog, in addition to providing a portable model.

Because FPGAs are not just processors using a typical software design flow, targeting FPGAs from OpenCL presents some special challenges. The FPGA architecture differs significantly from the typical platforms (such as CPUs and GPUs) that OpenCL implementations target. For instance, FPGA makers recently debuted programmable system-on-chips (SoCs), in which a SoC is connected to FPGA fabric to create a customizable platform for an embedded system environment, like the Zynq platform [17]. Additionally, there is plenty of room for OpenCL to adjust to this kind of platform due to the long compilation time, the programmable nature of FPGAs, and the capability for partial reconfiguration [18].

**Fig 2:**
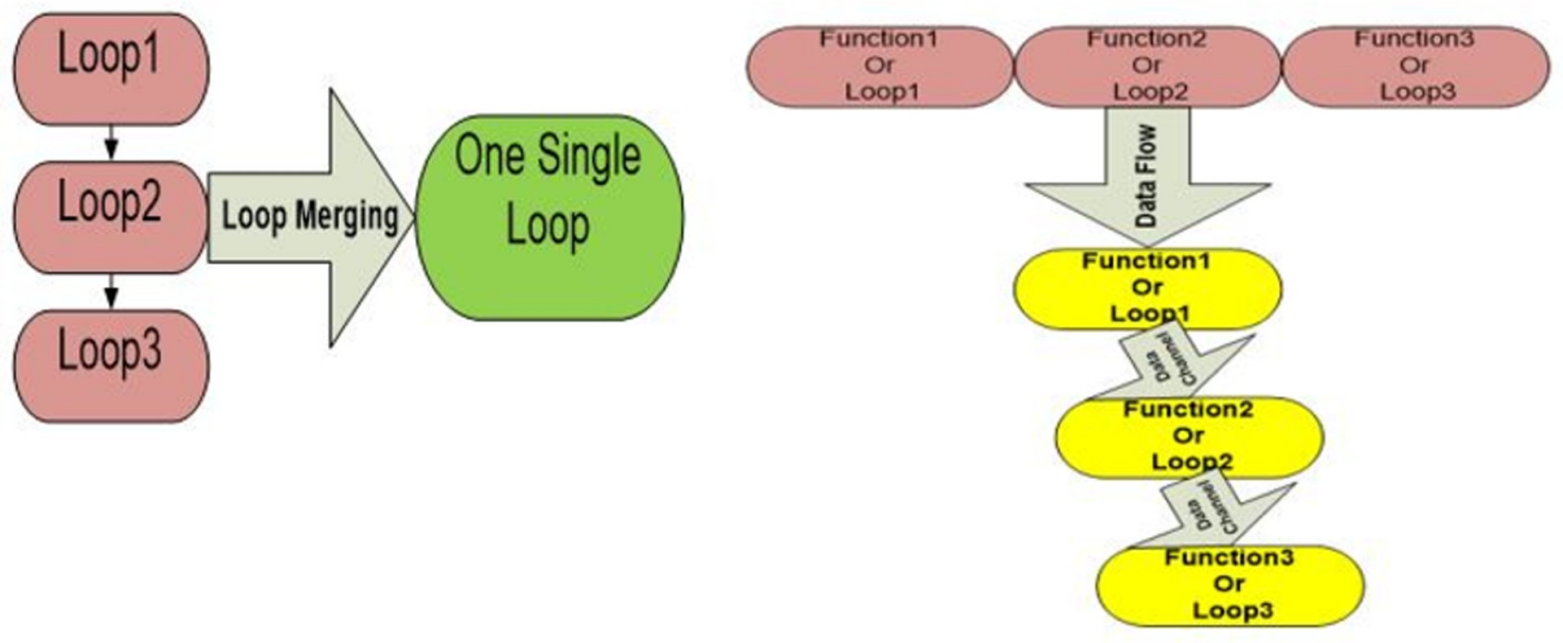
Examples of optimization techniques can be applied to the proposed design using the FPGA software development tool.

**Fig 3:**
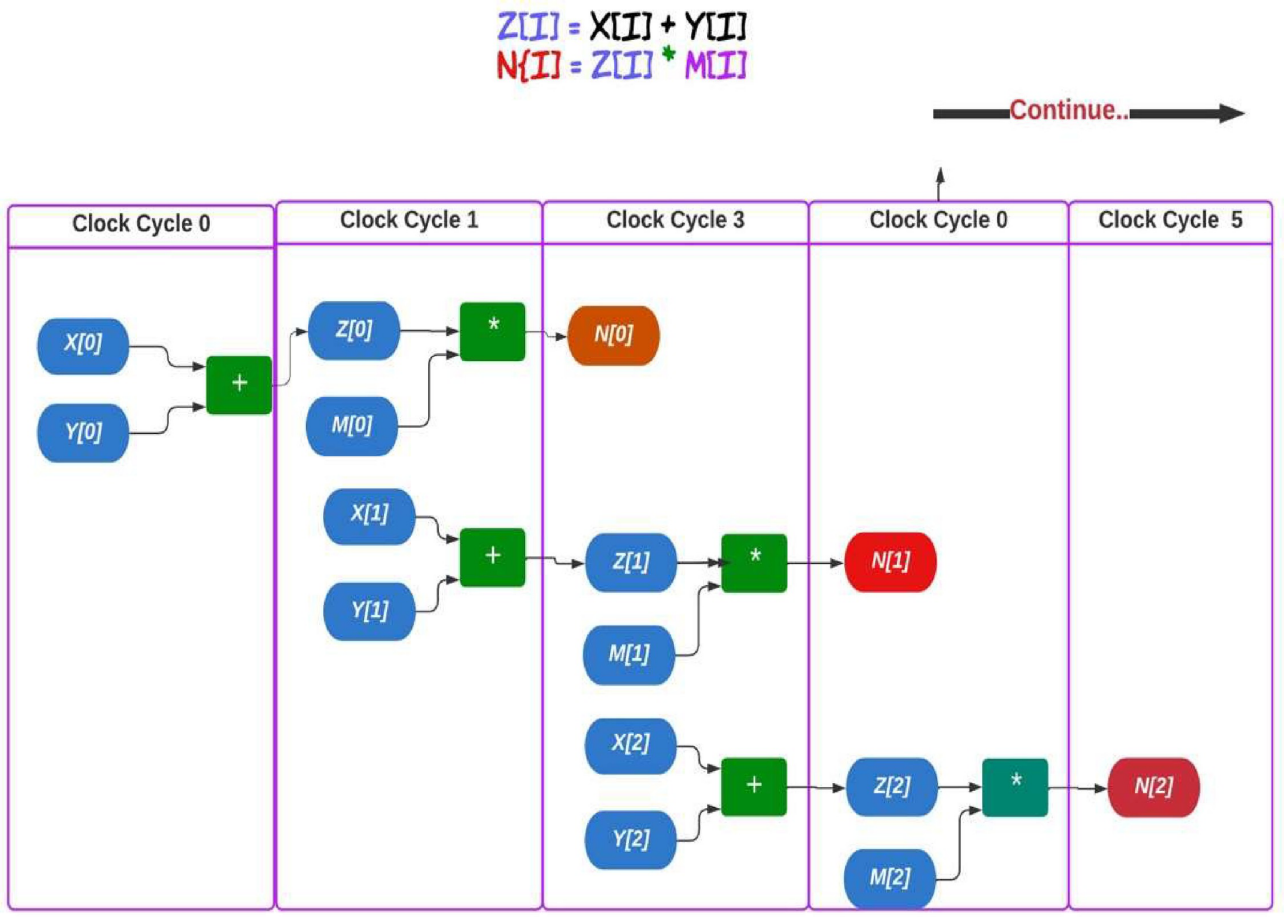
Illustration of the pipeline architecture created by the IOC for a given kernel code written in OpenCL.

The notion of pipeline parallelism is an important concept of the IOC, which synthesizes the high-level abstracted OpenCL code on the target FPGA device. Pipelined architectures allow data to pass through various stages before the proposed result is attained. The IOC creates a customized pipeline architecture based on the proposed kernel code [19]. Figs 2 and 3 illustrate how the IOC creates the pipeline architecture for a given kernel code. Several optimization techniques, such as shift-register, data flow, loop merging and loop unrolling can feasibly be used to create a powerful design architecture as shown in Figs 2 and 3. Loop unrolling allows more operations to be performed per clock cycle by duplicating the necessary function units. Meanwhile, the shift-register technique helps reduce the dependency between consecutive statements, thereby reducing the number of stall cycles. By incorporating data flow and loop merging, the proposed design gains more capability to overlap instruction execution. The Intel FPGA compiler provides several tools to modify the design’s performance and solve possible critical issues that may reduce the effectiveness of the architecture before synthesizing the proposed FPGA device [20].

The code written in OpenCL to perform the acceleration process on the Intel FPGA architecture has two parts. The first part is the host code, which is written in standard C/C++ code and compiled using the gcc/g++ compiler [8]. The host code is responsible for starting the acceleration process, deciding what data should be transferred between the host and the FPGA global memory, and deciding which parts of the code should be accelerated on the FPGA device. The overall host code is a sequence of steps taken before and after the kernel code is launched. The second part is the kernel code, which is implemented using the OpenCL APIs and compiled with the IOC to generate the device executable code [10]. Whenever the FPGA undergoes reprogramming, the newly constructed design replaces the old one. Employing the FPGA as the primary processing platform facilitates easy control over the number of concurrent tasks and the data to be stored in memory. In the envisioned design, all operations are executed exclusively on the FPGA development board. This encompasses accessing the FPGA memory system, executing the proposed benchmark, and ensuring that only one algorithm runs at any given time.

The Intel offline compiler, functioning as a high-level thesis compiler, produces multiple files during the compilation process. Notably, VHDL and Verilog files are generated among these, and they play a crucial role in constructing various functional units in the final proposed design. Despite this, the use of VHDL or Verilog is more resource efficient. The additional resources demanded by OpenCL primarily stem from the necessity to incorporate OpenCL-compliant support logic on the FPGA, contributing to a resource-area overhead.

This work is based on the study titled “Efficient Synchronization primitives for GPU” [Stuart and Owens 2011] [21], in which a set of eight benchmarks was developed using a CUDA software framework to study the effect of memory access time on overall performance. The present study intends to replicate this work and study the effect of several synchronization functions on the overall performance when targeting the general FPGA computing platform. Several synchronization techniques can be used when multiple threads cooperate to perform related tasks and have access to commonly shared variables. The barrier is a common synchronization technique that allows all synchronized threads to stop at a certain point; then, once the last thread reaches this point, all threads resume their execution [22]. Mutex is another synchronization mechanism that allows only one thread to execute in the critical section to avoid the race condition issue. A binary semaphore is similar to a mutex mechanism when a single resource exists [23]. Meanwhile, the counting semaphore controls access to shared resources when there are multiple instances of a resource, and each instance cannot be used by more than one thread concurrently [24].

Although these synchronization techniques must be used to control access to shared resources, they introduce a significant time overhead due to the overall waiting time required for each thread to access the critical resource [25]. The present research recommends a technique that could have less overhead than other techniques when implemented on the FPGA platform. Several benchmarks are developed to analyze the memory access time of various implementations, thereby achieving the study’s purpose. These benchmarks are classified as atomic or non-atomic in the first layer and as having high or low contention in the second layer.

Finally, for read or write memory operations, we define atomic access as the access of only one thread to a distinct memory location, such that atomic accesses to the same memory location must be serialized. High contention means that all threads will access the same memory location. Meanwhile, in low contention instances, we generate multiple memory locations separated by at least 64 bytes, and there is a minimal chance that two or more threads will access the same memory location [21]. Lastly, in this design, we assume that each workgroup has 128 work items. Only one thread (the master thread) is given access to memory; for simplicity, thread zero is the master thread.

All benchmarks are compiled using the GCC V4.4 and the Intel FPGA compiler V13.1, which are compatible with the Linux Centos operating system. The target FPGA used is the DE5 Stratix V device (5SGXEA7N2F45C2). This board contains enough resources, including 234K ALMs, more than 250 DSP blocks, and 2.6K RAM blocks to synthesize the user’s code in various heavy computation applications. The host CPU and the target board are connected via a (PCIe) connection, which enables extremely rapid data transfers between the processing units. The benchmarks created within the abstracted OpenCL framework are compatible with a wide range of FPGA types. For Xilinx FPGAs, the SDAccel framework, analogous to the Intel OpenCL framework tool, can be employed to execute various benchmarks on the designated Xilinx board.

## 2. Available resources vs. Throughput trade-offs

Each FPGA contains a countable number of specific resources, such as ALMs, memory blocks, and DSPs. The proposed FPGA is usually connected to the host machine using the PCIe interface [6]. Each kernel is translated into a proposed hardware circuit using a fixed amount of resources. Typically, all kernels are combined into a single.cl (device code) file. While it takes only microseconds to milliseconds to run the kernel on the proposed FPGA (depending on the synthesized design), the overhead associated with switching the kernel during runtime is extremely large. The experiment was run 100 times to determine that it took approximately 1.612 seconds to configure the device at runtime. This outcome indicates that the configuration time is significant in most cases.

Another factor to consider is the additional resource consumption associated with using a high-level abstract OpenCL programming tool. Experiments demonstrate that approximately 16% of the ALMs, 11% of memory blocks, 3% of the total memory bits, and 53,893 registers are consumed to implement a blank (empty code) kernel. The extra resource overhead shown in Table 1 can be avoided by combining multiple kernels into a single file. Table 1 summarizes multiple kernels of vector addition, where the kernel is duplicated up to five times in a single file. Column 2 shows the resource usage of the blank kernel; Column 3 shows the resource usage of a single vector addition kernel; and Columns 4–7 show the resource usage of two, three, four, and five vector addition kernels. The experiment demonstrates the overhead associated with using a high-level abstracted OpenCL tool.

**Table 1:** FPGA resource usage for a single and multible vector addition kernel.

Resources/kernel	Blank Kernel	Vadd-1 kernel	Vadd–two kernels	Vadd- three kernels	Vadd–four kernels	Vadd–five kernels
Logic utilization (ALMs)	16%	20%	23%	26%	29%	31%
Total registers	53,893	72,397	84,439	97,485	106,650	115,269
M20k blocks (RAM blocks)	11%	14.72%	17.38%	20%	21.68%	22.62%
Total block memory bits	3%	3%	3%	4%	4%	4%

Loop unrolling can enhance performance by running several loop iterations in each clock cycle. However, the duplication function unit required to implement the loop unrolling technique consumes more resources. As such, the loop unrolling factor depends mainly on the number of resources available. Because of hardware limitations, we cannot fully unroll the loop in this work. Therefore, the loops in all benchmarks are unrolled 256 times. The same is true for different mutex implementations; all implementations are unrolled 10 times.

## 3. Proposed method and the developed benchmarks

**Fig 4:**
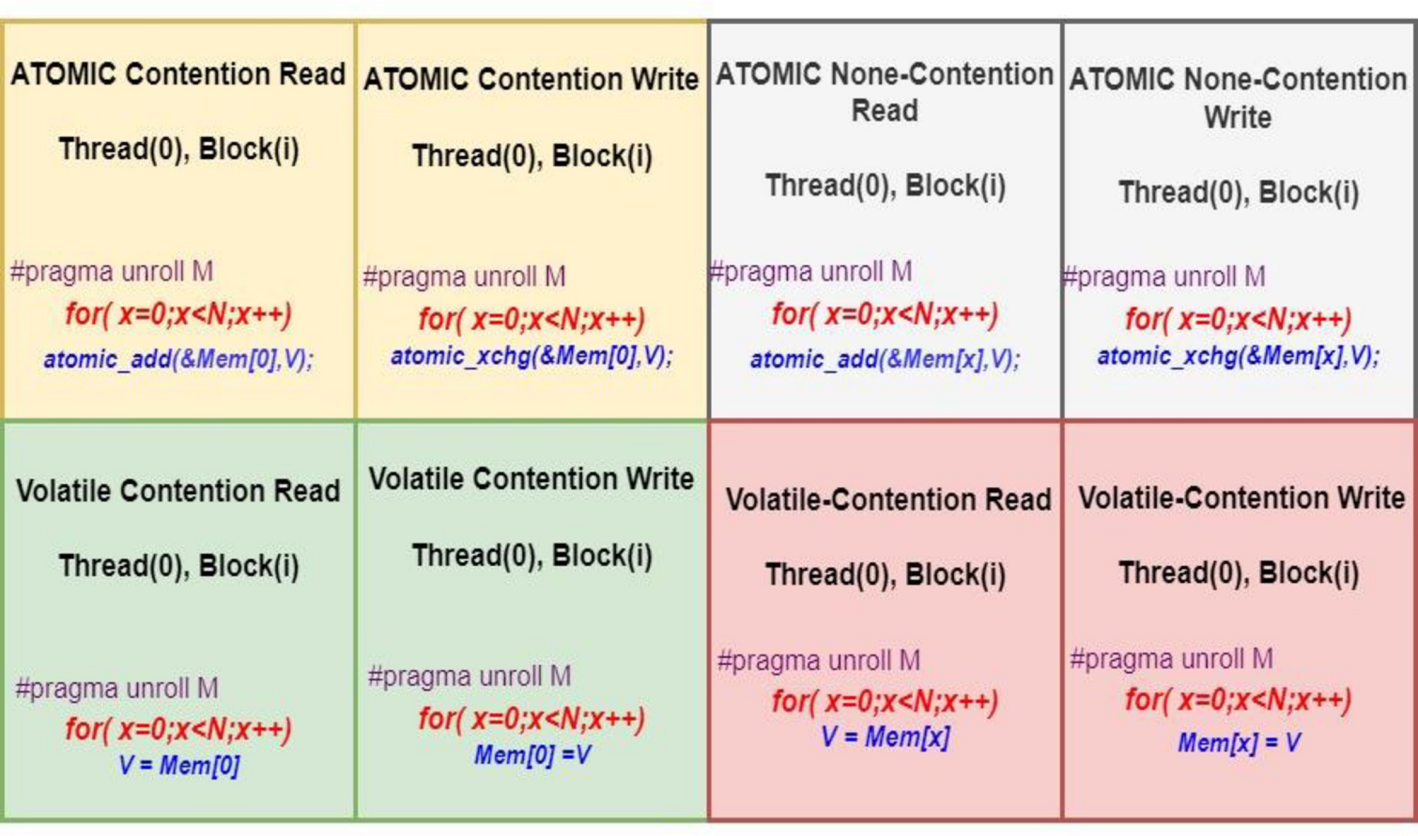
Overview of benchmarks created for assessing FPGA memory access time.

A set of eight benchmarks is created and compiled with the IOC to test the performance of FPGA memory systems. The benchmarks are classified as atomic or non-atomic, contentious or non-contentious, and read or write. For an atomic memory access operation, only one thread can access the desired memory location at a time, and no other thread can access the same memory location concurrently. In cases of contentious access, all threads access the same memory location, whereas in non-contentious access cases, different threads access different memory locations. Threads are divided into work groups, each of which contains 128 threads. However, only one thread in each workgroup (the first and master thread) can access the memory. Each master thread performs 1024 memory access operations, which can be read or written. The *“atomicadd”* operation is used to implement the atomic read, and *“atomicexchange”* is used to implement the write operation. All benchmark loops are unrolled 256 times; this unrolling factor number is based on the available resources on the target FPGA to synthesize the proposed design. Fig 4 below summaries the developed benchmarks.

Certainly, these benchmarks serve as representative indicators and encompass various memory access behaviors. The primary concern addressed here is memory access time, particularly crucial when targeting parallel and high-speed computation platforms like GPUs, multi-cores, and FPGAs. Numerous researchers have delved into discussions regarding memory access time and synchronization issues in the context of GPUs and multi-core computation platforms. This study is introduced to specifically investigate the FPGA memory system and its impact on overall FPGA performance in relation to memory access time.

Table 2 shows that atomic operations need more time to execute. Reducing the number of atomic operations will enhance the performance significantly. The effects of contention are not marked. The computing unit is saturated by running eight workgroups, each comprising 128 threads. Only the master thread can access the desired memory location. Certainly, this study considers various workloads and data sizes. The performance of these algorithms is evaluated through a set of experiments involving different numbers of thread blocks and varying numbers of memory operations.

**Table 2:** Benchmarks execution times for 1000 memory operations, measured in milliseconds.

	Read (ms)	Write (ms)	Average access time (ms)
Atomic contention	545	569	557
Atomic non-contention	422	428	425
Contention volatile	64	71	68
Non-contention volatile	68	73	71

**Fig 5:**
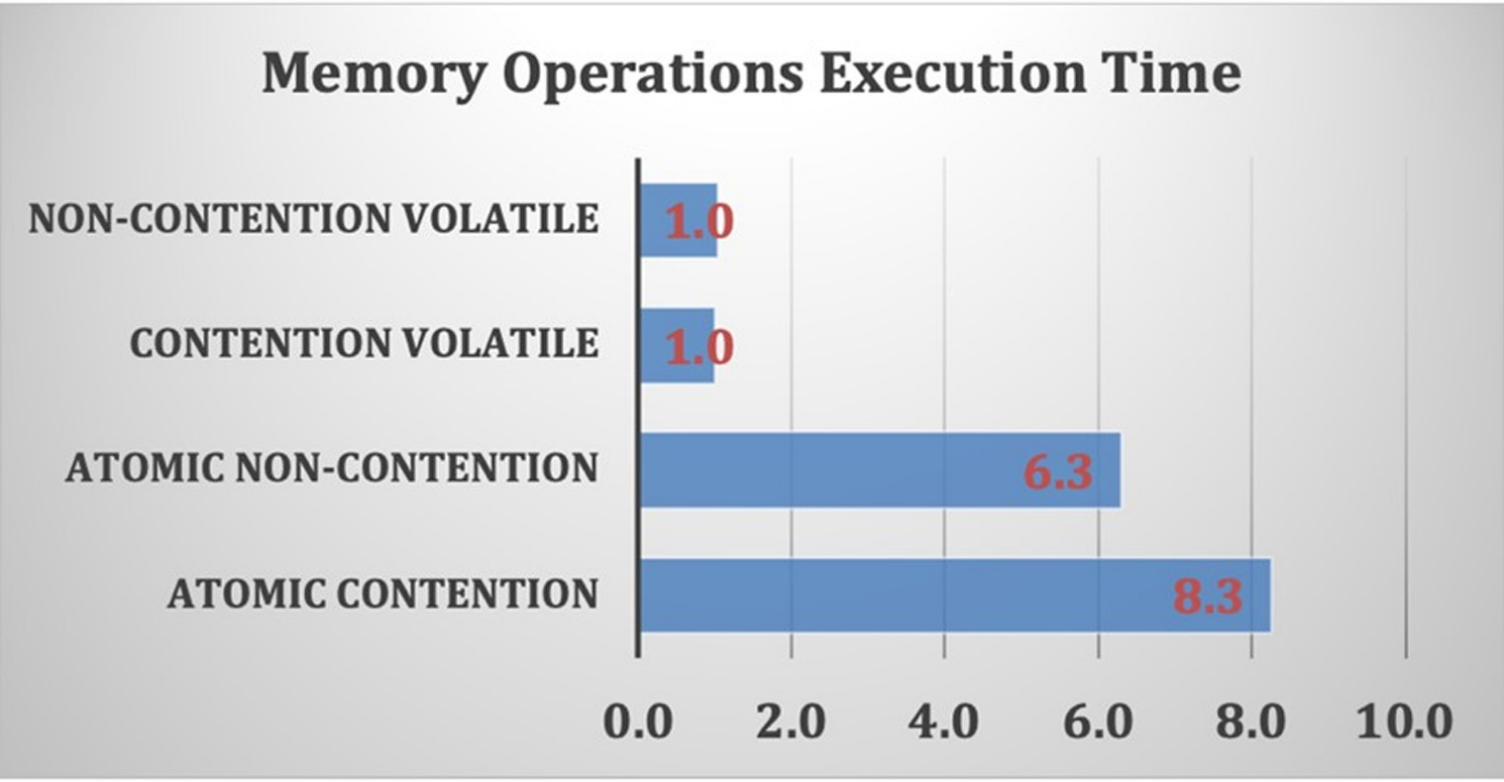
Average memory execution times normalized to the execution time of a contention volatile memory operation.

The average execution times of various memory read/write operations are shown in Fig 5, and these are normalized to the execution time of a contention volatile memory operation. Fig 5 also shows the effects of atomic operations on memory access operations, which may increase the memory access time by more than eight times. However, the task-parallel model is more commonly used to construct the proposed design on the FPGA platform. The Intel FPGA compiler has the capability to create an effective pipeline design where data can be shared among multiple loop iterations; this reduces the overall dependencies and the high cost of using several synchronization mechanisms.

## 4. MUTEX implementation and results discussion

After studying memory access benchmarks, several possible implementations of mutex are developed and tested on the Intel FPGA architecture. All proposed algorithms perform atomic memory access, and only the master thread has access to memory. These suggested implementations [21] are described below.

A. Spinning: in this implementation, the target thread is in the waiting state until the status of the proposed memory location is changed. Two operations are considered here:
  - *Lock function*: The memory location is continually accessed using *atomicexchange*, which always returns the old value of the lock. If the returned value of the lock is 0, the thread can access the critical section; otherwise, it will continuously perform *atomicexchange* until it is granted access to the critical section.
  - *Unlock function*: The critical section is released by assigning a lock value of 0 using *atomicexchange*. This method is easy to implement; however, threads are not necessary to access critical sections in the same order in which they arrive (not FCFS).
B. Backoff: the target thread continues doing non-useful work before getting access to the resource, two operations are carried out:
  - *Lock function*: The thread tries to gain access to the critical section if it is free. Otherwise, the thread sleeps for a certain time based on the thread group ID. This time increases after each trial until it reaches the maximum value, which is determined during the compilation time. This value is assigned to the minimum value if the incremented value is greater than the maximum value. This process is repeated until the thread accesses the critical section.
  - *Unlock function*: The unlock function assigns a lock value of 0 (nonatomic operation).
C. Fetch and add using Backoff: A well-known instruction supported by many processors to introduce an effective mutex implementation. The Backoff is employed here to let the thread wait if the resource is not available, two operations are implemented here:
  - *Lock function*: Each thread that should gain access to the critical section takes a ticket (the first variable), which is a number based on the thread’s arrival order. The thread can access the critical section only if the value of the ticket is equal to the value of the turn (the second variable). If the ticket value is not equal to the turn value, the thread uses the Backoff algorithm to sleep for a certain period of time.
  - *Unlock function*: Increment the turn value (nonatomic operation).
D. Fetch and add using sleeping: Same as in "Fetch and add using Backoff", but with the sleeping technique is used instead of backoff to implement the thread waiting.
  -*Lock function*: This function is the same as that described in the Fetch and add using the Backoff algorithm, but if the ticket value is not equal to the turn value, the thread continuously polls the variables’ memory locations to check if the equality condition is satisfied.
  - *Unlock function*: Increment the turn value (nonatomic operation).

**Fig 6:**
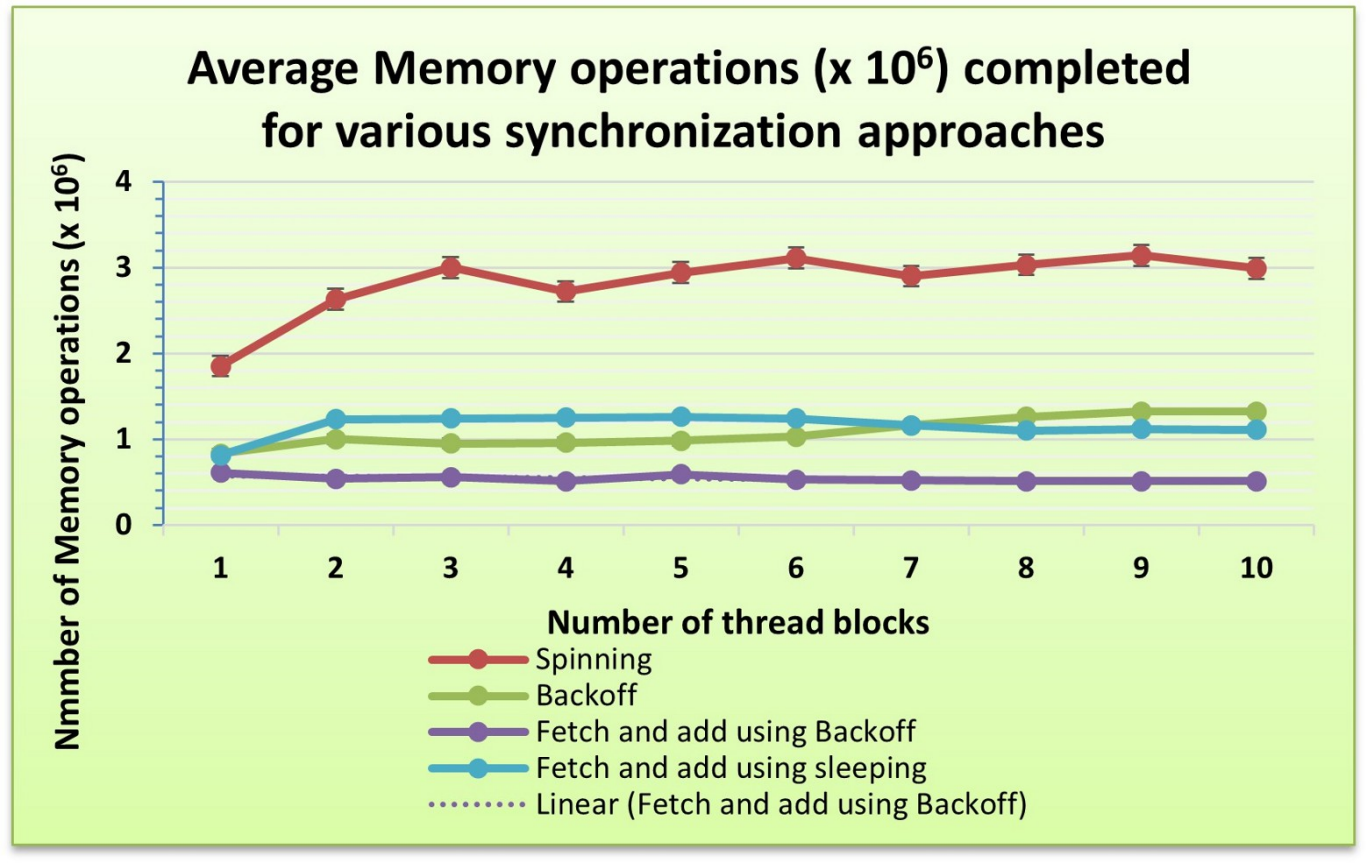
A comparative analysis of performance among various synchronization implementations.

Several experiments with varying numbers of thread blocks are carried out to compare the performance of these algorithms. The performance of each algorithm is evaluated by measuring the number of memory operations completed per second. Table 3 shows the experimental results, which demonstrate that the highest throughput is achieved using the spinning implementation of mutex, as shown also in Fig 6. Values represent millions of memory operations per second on the Intel DE5 FPGA device. In this case, the target platform is a general Intel FPGA device.

**Table 3:** The number of operations completed per second (x 10^6^). Spinlock is the preferred implementation, and the Fetch and Add using the Backoff algorithm has the lowest throughput.

Number of blocks	Spinning	Backoff	Fetch and add using Backoff	Fetch and add using sleeping
1	1.85	0.83	0.61	0.81
2	2.63	1.00	0.54	1.23
3	3.00	0.95	0.56	1.24
4	2.72	0.96	0.51	1.25
5	2.94	0.98	0.59	1.26
6	3.11	1.03	0.53	1.24
7	2.90	1.16	0.52	1.16
8	3.03	1.26	0.51	1.10
9	3.14	1.32	0.51	1.12
10	2.99	1.32	0.51	1.11

The preferred implementation is that which uses the fewest hardware resources. Table 4 shows some common resources used for each algorithm. The proposed synthesized architecture of the spinning algorithm consumes fewer resources than other algorithms. For all algorithms, each loop iteration contains 100 memory operations, and each operation has lock and unlock functions.

**Table 4:** Common hardware recourses used in each algorithm.

	Spinning	Backoff	Fetch and add using Backoff	Fetch and add using sleeping
LUTs (using parentage)	25%	67%	65%	38%
Registers	86.2 K	300.7 K	305.8 K	143.4 K
Total memory blocks (using parentage)	4%	12%	18%	5%

Applying synchronization methods like the Adaptive Distributed Consensus Control of One-sided Lipschitz Nonlinear Multiagent [26] and the Delay-range-dependent Chaos Synchronization approach, which considers varying time-lags and delayed nonlinear coupling [27], can be employed to investigate and analyze memory synchronization across various computation platforms. This necessitates the regeneration and development of new benchmarks. These novel approaches may be embraced in future studies, allowing for a comparison with the presented results.

## 5. Conclusion

Several memory-access-based benchmarks are developed to study the effect of common synchronization techniques on the overall performance of the proposed synthesized design constructed on the Intel FPGA platform. These benchmarks are developed using the abstracted high-level OpenCL programming tool. The results demonstrate that using atomic operations in the synthesized design leads to significant reductions in performance. Therefore, the task-parallel model, which improves the efficiency of the created design by generating an effective pipeline architecture, is a favorable choice when extra atomic operations are used. The present study also investigates several implementations of the widely used mutex synchronization mechanism and determines which implementation could be adopted by the proposed design to maximize the number of memory operations performed per second.
